# Association between social jetlag and the sleep regularity index in young adults with and without sleep disorders

**DOI:** 10.1093/sleepadvances/zpag102

**Published:** 2026-09-12

**Authors:** Katrina Nguyen, Nicole Lovato, Gorica Micic, Claire Dunbar, Josh Fitton, Gemma Robertson, Ashley Montero, Kelly A Loffler, Kelly Sansom, Andrew Vakulin, Robert J Adams, Andrew J K Phillips, Amy C Reynolds

**Affiliations:** Flinders Health and Medical Research Institute (Sleep Health), College of Medicine and Public Health, Flinders University, Adelaide, SA, Australia; Flinders Health and Medical Research Institute (Sleep Health), College of Medicine and Public Health, Flinders University, Adelaide, SA, Australia; AISH Sleep Health Clinics, College of Medicine and Public Health, Adelaide, SA, Australia; Flinders Health and Medical Research Institute (Sleep Health), College of Medicine and Public Health, Flinders University, Adelaide, SA, Australia; AISH Sleep Health Clinics, College of Medicine and Public Health, Adelaide, SA, Australia; Flinders Health and Medical Research Institute (Sleep Health), College of Medicine and Public Health, Flinders University, Adelaide, SA, Australia; AISH Sleep Health Clinics, College of Medicine and Public Health, Adelaide, SA, Australia; Flinders Health and Medical Research Institute (Sleep Health), College of Medicine and Public Health, Flinders University, Adelaide, SA, Australia; Flinders Health and Medical Research Institute (Sleep Health), College of Medicine and Public Health, Flinders University, Adelaide, SA, Australia; Flinders Health and Medical Research Institute (Sleep Health), College of Medicine and Public Health, Flinders University, Adelaide, SA, Australia; Flinders Health and Medical Research Institute, Flinders Clinical Trials, Flinders University, Adelaide, SA, Australia; Flinders Health and Medical Research Institute (Sleep Health), College of Medicine and Public Health, Flinders University, Adelaide, SA, Australia; Centre for Healthy Ageing, Murdoch University, Perth, WA, Australia; Flinders Health and Medical Research Institute (Sleep Health), College of Medicine and Public Health, Flinders University, Adelaide, SA, Australia; Flinders Health and Medical Research Institute (Sleep Health), College of Medicine and Public Health, Flinders University, Adelaide, SA, Australia; AISH Sleep Health Clinics, College of Medicine and Public Health, Adelaide, SA, Australia; Flinders Health and Medical Research Institute (Sleep Health), College of Medicine and Public Health, Flinders University, Adelaide, SA, Australia; Flinders Health and Medical Research Institute (Sleep Health), College of Medicine and Public Health, Flinders University, Adelaide, SA, Australia; AISH Sleep Health Clinics, College of Medicine and Public Health, Adelaide, SA, Australia

**Keywords:** circadian rhythm disorders, insomnia, insomnia – comorbid, OSA, statistics, biological rhythms

## Introduction

Sleep regularity, defined as the day-to-day consistency in sleep–wake patterns, is an integral component of sleep and overall health [1]. Lower sleep regularity (i.e. more irregular sleep) is associated with adverse health and performance outcomes [2], and recent research suggests that sleep regularity is a better predictor of mortality than sleep duration [3].

Although there are multiple available methods to quantify various aspects of sleep regularity, the most appropriate metric depends largely upon the specific study design/question and type of data [4]. To capture variation in sleep–wake patterns between consecutive days, Phillips et al. devised the Sleep Regularity Index (SRI), which quantifies the likelihood of being in the same state (asleep versus awake) at any two time-points 24 hours apart [5]. The SRI is intended to capture irregular sleep schedules and fragmented sleep patterns that can cause circadian disruption. SRI scores typically range from 0 to 100, with higher SRI scores indicating more regular sleep.

A related, but independent, construct is social jetlag, which reflects the discrepancy between sleep–wake patterns on weekdays versus weekends (or workdays versus work-free days) [6]. Social jetlag is typically measured by taking the difference in midsleep timing between workdays and work-free days, with higher values indicating greater social jetlag and less regular sleep. Social jetlag is more prominent in late chronotypes, who tend to have later bedtimes and therefore must curtail sleep to attend morning commitments. They often compensate for sleep loss accumulated during workdays by sleeping in later on their work-free days [7]. Social jetlag is especially prevalent among young adults, who often need to balance work or study obligations with domestic responsibilities or social/recreational activities [8].

Social jetlag [6] and the SRI [5] are two popular metrics for characterizing sleep regularity, but the extent to which they relate to one another has not yet been directly investigated. Although social jetlag could be assumed as a primary contributor to irregular sleep schedules in young adults, to date, no studies have specifically investigated the relationship between social jetlag and sleep regularity to answer whether social jetlag can serve as a practical proxy for sleep regularity in clinical populations. While social jetlag may contribute to low sleep regularity for certain groups, such as school students, this is unlikely to apply to all populations. Notably, social jetlag compares average sleep timing between workdays and work-free days, unlike the SRI, which captures daily, rapid variations in sleep timing [4].

To our knowledge, no previous study has directly quantified the relationship between social jetlag and SRI using sleep–wake patterns of real human participants, including those with sleep disorders. Fischer et al. [4] compared social jetlag and the SRI using simulated sleep–wake patterns, showing that the two metrics measure different aspects of regularity. Our study extends previous work by using data from young adults with and without sleep disorders to examine whether the theoretical divergence identified by Fischer et al. is observed in sleep–wake schedules, which can be affected by factors such as fragmented and disordered sleep, napping, and shift work that are not featured in simulated schedules or those of healthy subjects*.* These factors could influence the association between social jetlag and SRI compared to simulation-derived predictions.

To address this knowledge gap, we aimed to determine the variance in SRI explained by social jetlag among young adults with and without clinically diagnosed sleep disorders. By examining both young adults with and without sleep disorders, we will also determine whether the association between the metrics differs based on clinical status. This approach allows us to explore whether the overlap between the metrics is consistent across clinical and non-clinical samples, with translational implications for how sleep regularity should be measured in clinical populations.

## Materials and methods

Our sample consisted of young adults with and without sleep disorders (age range: 18–38 years) who were enrolled in a clinical trial (ACTRN12624000339550) that aimed to investigate whether the combination of sleep education and an in-laboratory shift-work simulation could improve help-seeking for sleep problems among future healthcare workers [9]. Participants were enrolled in medicine, paramedicine, or nursing degree sequences, and were invited to complete screening questionnaires and a sleep disorder education module as part of the first trial component. Those who consented to participate in the in-laboratory shift-work simulation trial component, and were screened as eligible, came into the laboratory for a 2-hour screening session, after which they completed 1–3 weeks of sleep diaries.

Individuals were included if they reported no illicit drug or smoking use that meant they could not abstain for the duration of the in-laboratory protocol; were not pregnant, lactating, or caring for a newborn (<12 months); did not report unmanaged suicidal ideation or intent; and did not have a history of traumatic brain injury, stroke, or other neurodegenerative disorders.

Participants did not have an existing sleep disorder diagnosis before entering the trial. Diagnoses were either confirmed as part of the trial procedure (see Dunbar et al. [9]) using validated screening questionnaires and a structured clinical interview administered by a sleep psychologist for those with sleep disorders, or ruled out if they did not meet diagnostic criteria for a sleep disorder (classified in these analyses as “healthy sleepers”).

### SRI and social jetlag calculations

Participants recorded their sleep–wake timings using daily sleep diaries prior to diagnosis (range of use: 1–3 weeks, median [Interquartile range] days of use = 21 [4]). Based on previous work measuring SRI [10], including the article in which SRI was introduced and validated [5], 7 days of diary data meet the minimum number of days of overlapping data to calculate valid SRI scores. Any wake state reported in each diary entry was coded as “0” and any sleep state as “1.” The SRI is calculated based on the percentage likelihood that the individual is in the same state (asleep or awake) at two time-points 24 hours apart, averaged across the number of recording days. The calculation involves comparing time-matched epochs 24 hours apart, dividing the number of matching states by the total number of comparisons and scaling to a 0–100 scale, where 0 indicates completely random sleep–wake patterns and 100 completely identical sleep–wake patterns on consecutive days. Social jetlag was derived from responses to the Munich Chronotype Questionnaire (MCTQ) [7], which participants completed on the evening of an in-laboratory stay, calculated as the absolute difference in midsleep point timing between workdays (MSW) and work-free days (MSF): SJL = |MSF – MSW|, where midsleep is calculated as (Bedtime + Wake-up time)/2 [6].

### Statistical analysis

Separate multiple linear regression models were performed for individuals with (“sleep disorder”) and without sleep disorders (“healthy sleepers”) in RStudio with R version 4.4.2 (see Supplementary S1). Data were analyzed and plotted to compare the variance in SRI explained by social jetlag across the two groups.

## Results

Due to the nature of the trial, the sample was enriched for sleep disorders, meaning 82% met criteria for one or more sleep disorders (*n* = 61, 59% female; age [mean ± SD] = 25.3 ± 5.1 years), while 18% were considered “healthy sleepers” (*n* = 13, 54% female; age [mean ± SD] = 26.1 ± 4.7 years) and retained for comparison purposes. Among those with sleep disorders, diagnoses included Insomnia (43%), Delayed Sleep–Wake Phase Disorder (DSWPD) (33%), Obstructive Sleep Apnoea (31%), Excessive Daytime Sleepiness (11%), Insufficient Sleep Syndrome (6%), and other (15%; noting totals >100 % due to co-morbidities). 39 per cent of participants with sleep disorders had a comorbid sleep disorder presentation. Participants with sleep disorders and the healthy sleepers, respectively, did not differ in median age (25.0 [21.0, 28.0] years; 25.0 [23.0, 28.0] years), sex distribution (36 [59 per cent]; 7 [54 per cent] female), or median habitual sleep duration (7.2 hour [6.7, 7.6]; 7.5 hour [6.9, 7.9]), which was calculated from their sleep diary data.

In the sleep-disordered group, higher social jetlag was significantly associated with lower SRI (*β* = −5.01, SE = 1.21, *p* < .001) (Figure 1). Social jetlag explained 21% of the variance in SRI (adjusted *R*^2^ = 0.21), and the overall model was statistically significant, *F*(1,59) = 17.1, *p* < .001. Adjusting for sleep duration did not meaningfully change the variance explained by social jetlag (data not shown). There were outliers in our analyses, which were retained as they represented genuine variability in sleep regularity. When removed from the analyses for conceptual comparison, variance in SRI explained by social jetlag dropped to 8% (*R^2^* = 0.093; adjusted *R^2^* = 0.077), *F*(1,57) = 5.82, *p* = .02. These cases highlight that social jetlag may variably explain variance in sleep regularity according to SRI and highlight the importance of considering additional contributing factors in the context of a sleep disorder presentation. In the healthy sleepers, higher social jetlag was associated with lower SRI, but this did not reach statistical significance (*β* = −4.28, SE = 2.05, *p* = .061). Social jetlag explained 22% of the variance in SRI (adjusted *R^2^* = 0.22), with a non-significant overall model fit, *F*(1,11) = 4.37, *p* = .061.

**Figure 1:**
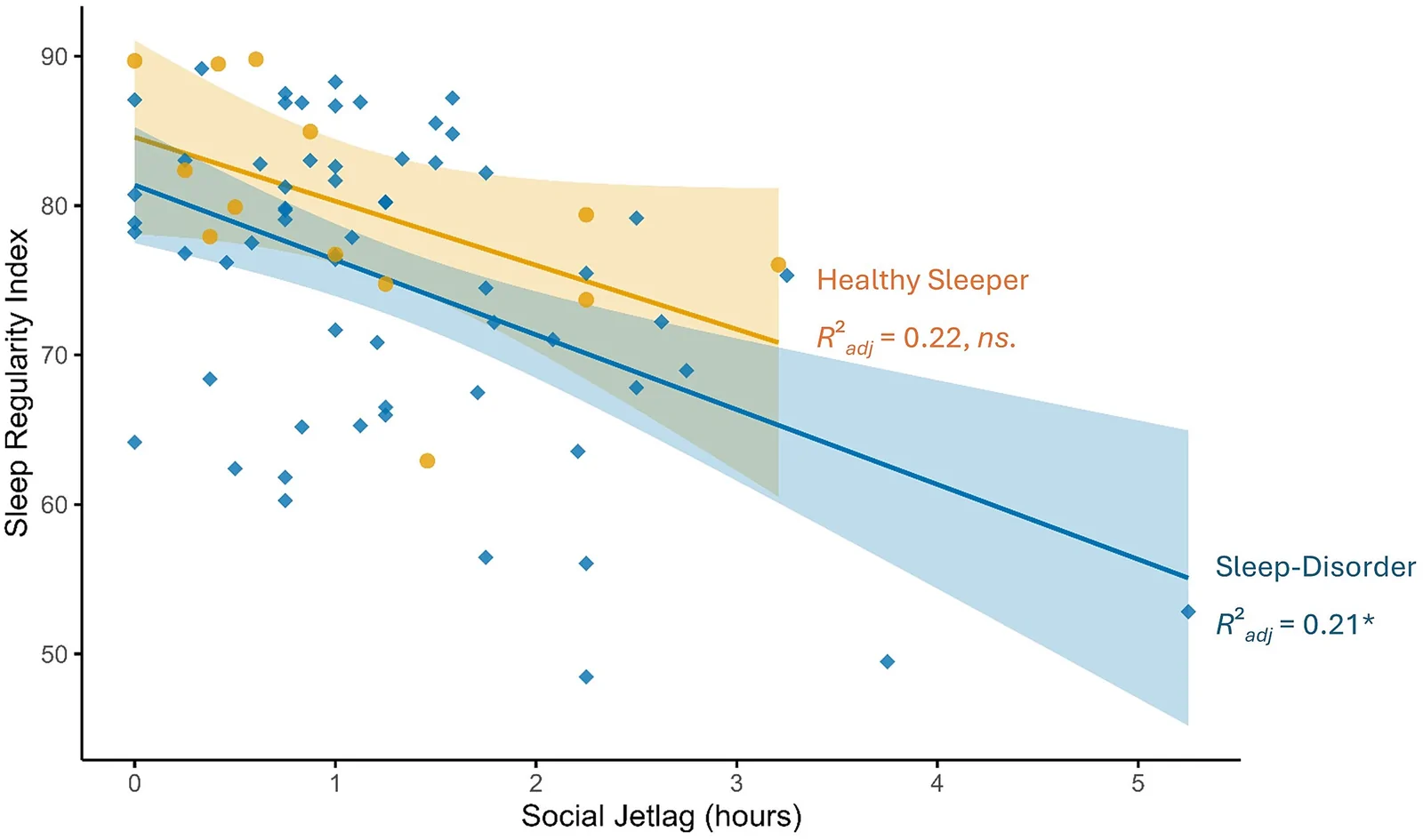
Relationship between social jetlag and SRI in the sleep disorder and healthy sleeper groups. Each point represents an individual participant (Circle = healthy sleeper, diamond = sleep disorder). Linear regression lines represent separate within-group fits for each group, with shaded areas indicating the 95% confidence intervals. Reported *p*-values represent the significance of the within-group regressions (*, p < .001; ns, not significant).

## Discussion

Greater social jetlag was associated with lower sleep regularity in young adults with sleep disorders, independent of habitual sleep duration. While social jetlag contributed to irregular sleep, it explained only 21% of the variance in SRI in this population. The modest association between social jetlag and SRI is consistent with these two metrics capturing related but distinct aspects of sleep regularity, suggesting they should not be assumed to be interchangeable. The non-significant association between social jetlag and SRI in the healthy sleepers likely reflects limited power with a smaller sample size, as the direction of the association was consistent with the pattern observed in the sleep-disordered group. These findings in the smaller healthy sleeper subgroup provide preliminary evidence on the consistency of the observed association between social jetlag and SRI across groups with and without sleep disorders, and these preliminary findings should be validated in larger, adequately powered samples.

Our findings align with Fischer et al.’s simulation article comparing multiple sleep regularity metrics [4], including social jetlag and SRI, which demonstrated that these metrics are correlated but not equivalent. The current study adds to this knowledge by empirically examining whether the partial correlation observed by Fischer et al. is observed in real sleep–wake schedules from a clinical population. Factors influencing sleep–wake schedules such as fragmented sleep, napping, and shift work could have influenced the association between social jetlag and SRI relative to simulation-derived estimates.

Our study used two data sources (MCTQ for social jetlag; sleep diaries for sleep regularity), meaning we cannot rule out the possibility that the variance unexplained reflects methodological differences rather than distinct underlying constructs. We could not assume workdays relative to free days in the sleep diaries, given the propensity for non-standard work hours and placement schedules in young adults undertaking healthcare degrees. Therefore, while we can speculate that social jetlag contributes to, but does not fully explain, SRI in young adults with sleep disorders, future research that derives sleep regularity across multiple metrics and over time will support more definitive conclusions.

Furthermore, as insomnia and DSWPD constitute a large proportion of the sleep disorder composition (43% and 33% respectively among those with sleep disorders, including those with comorbid disorders), these disorders may contribute to the observed associations as they may influence sleep regularity directly in distinct ways. Variability in sleep–wake patterns due to intermittent awakenings and variable sleep onset latency may be characteristic of insomnia [11], which may reduce SRI but not necessarily increase social jetlag. Meanwhile, DSWPD could be associated with greater social jetlag due to discrepancies in sleep–wake timings on work-free days versus workdays, while not necessarily reducing SRI. Therefore, the observed association between the metrics may in part reflect the specific sleep disorder composition of our sample and may not generalize to clinical populations with a different composition.

Nevertheless, other factors likely contribute to irregular sleep in this population beyond social jetlag, including variation in sleep timing within workdays and within work-free days. Future research into psychosocial and behavioral factors that predict irregular sleep in young adults with sleep disorders is warranted. For example, lifestyle factors such as shift work [12] and mental [13, 14] and physical health status [15, 16], and modifiable behavioral factors such as sleep parameters (e.g. time taken to fall asleep, napping behavior) may better explain SRI in young adults, rather than discrepancies in sleep–wake timings between weekdays and weekends, which are a common assumption.

Future research could also investigate weekend catch-up sleep (increased total sleep time on the weekend) alongside social jetlag to better characterize weekday–weekend discrepancies in sleep, given that social jetlag captures delays in sleep periods, but not recovery sleep. Our exploratory findings are consistent with social jetlag and SRI appearing to capture related, but different aspects of sleep timing and regularity, and we speculate that various biopsychosocial factors likely influence this relationship.

## Supplementary Material

Linear_regression_model_r_code_zpag102

## Data Availability

The data presented in this article may be shared on reasonable request to the corresponding author, subject to approval from the governing ethics committee.
