# Supplementary material for: Association between social jetlag and the sleep regularity index in young adults with and without sleep disorders
Source: Sleep Adv. 2026 Sep 12;7(4):zpag102. doi: 10.1093/sleepadvances/zpag102 (PMC13660469; doi:10.1093/sleepadvances/zpag102)
Supplement: Supplementary file 1 [file linear_regression_model_r_code_zpag102.docx]

\# Linear regression model code

df_control <- SJLvSRIFULL %>% filter(Primary == "Control")

df_sleepdis <- SJLvSRIFULL %>% filter(Primary == "SleepDisorder")

model_control <- lm(SRI ~ SJL_hours, data = df_control)

model_sleepdis <- lm(SRI ~ SJL_hours, data = df_sleepdis)

r2_control <- summary(model_control)$r.squared

r2_sleepdis <- summary(model_sleepdis)$r.squared

adj_r2_control <- summary(model_control)$adj.r.squared

adj_r2_sleepdis <- summary(model_sleepdis)$adj.r.squared

r2_df <- data.frame(

Group = c("Control", "Sleep Disorder"),

R2 = c(r2_control, r2_sleepdis),

Adj_R2 = c(adj_r2_control, adj_r2_sleepdis)

)
